# *Trichinella spiralis*: Knockdown of gamma interferon inducible lysosomal thiol reductase (GILT) results in the reduction of worm burden

**DOI:** 10.1371/journal.pntd.0009958

**Published:** 2021-11-30

**Authors:** Hong Fei, Muhammad Ali-ul-Husnain Naqvi, Sana Zahra Naqvi, Lixin Xu, Xiaokai Song, Xiangrui Li, Ruofeng Yan

**Affiliations:** MOE Joint International Research Laboratory of Animal Health and Food Safety, College of Veterinary Medicine, Nanjing Agricultural University, Nanjing, China; University of Passo Fundo: Universidade de Passo Fundo, BRAZIL

## Abstract

*Trichinella spiralis* is mammalian skeletal muscles parasite which may cause trichinellosis in animals and humans. Gamma interferon inducible lysosomal thiol reductase (GILT) is a widespread superfamily which plays key role in processing and presentation of MHC class II restricted antigen by catalyzing disulfide bond reduction. There are no reports about GILT in *T*. *spiralis*. In present study, GILT from *T*. *spiralis* (Tsp-GILT) was cloned, analyzed by multiple-sequence alignment, and predicted by 3D structure model. Recombinant Tsp-GILT (about 46 kDa) was efficiently expressed in *Escherichia coli* and thiol reductase activity suggested that in acidic environment the addition of a reducing agent is needed. Soaking method was used to knockdown expression of Tsp-GILT using small interference RNA (siRNA). Immunofluorescence assay confirmed the transformation of siRNA into muscle larva (ML) and new born larva (NBL). Quantitative real time-PCR (QRT-PCR) analysis revealed that transcription level of Tsp-GILT mRNA can be up-regulated by stimulation of mouse IFN-γ and down-regulated by siRNA2 *in vitro*. NBLs soaked with siRNA2 showed 32.3% reduction in the generation of MLs. MLs soaked with siRNA2 showed 26.2% reduction in the next generation of MLs, but no significant effect was observed on adult worms or NBLs. These findings concluded that GILT may play important roles in the development of *T*. *spiralis* parasite.

## Author summary

*Trichinella spiralis* is the most important pathogen causing trichinellosis of animals and humans. Reports in mammal animals showed that gamma interferon inducible lysosomal thiol reductase (GILT) play key roles in processing and presentation of MHC class II restricted antigens. However, there were no reports about GILT in nematode *T*. *spiralis*. In this study, GILT gene from *T*. *spiralis* (*Tsp-GILT*) was cloned and analyzed. The predicted 3D structure of Ts-GILT protein was found similar with that of *Homo sapiens* and *Sus scrofa*. To further evaluate the roles of *Tsp-GILT* in *T*. *spiralis* development, a soaking method was used to knockdown the expression of *Tsp-GILT* gene by siRNA. It was found that NBLs soaked with siRNA showed 32.3% reduction in the generation of MLs, and MLs soaked with siRNA showed 26.2% reduction in the next generation of MLs. Current study concluded that siRNA knockdown of gamma interferon inducible lysosomal thiol reductase from *Trichinella spiralis* significantly reduce the larval infectivity, development and survival.

## 1. Background

RNA interference (RNAi) is an important tool to determine the role of genes in which double-stranded RNA or small interfering RNA (siRNA) activates the breakdown of homologous mRNA. RNAi was firstly discovered in the *Caenorhabditis elegans*, nematode worm [1] and it has been broadly used to down-regulate target molecules by reducing mRNA for protein expression. Gene knockdown technique has been practiced efficiently in parasites by designing and delivery of target molecules. In previous studies, RNAi has been used to evaluate the biological functions of different genes in different helminthes, for example paramyosin in *T*. *spiralis* [2], type V collagen and calcium-regulated heat-stable protein in *Schistosoma japonicum* [3,4] and enolase in *Clonorchis sinensis* [5].

*Trichinella spiralis* is a tissue-dwelling parasite of mammalian skeletal muscles that may cause trichinellosis in more than 150 species of animals and humans [6]. Infection is acquired by taking raw or undercooked meat from where muscle larva (ML) enter the host’s small intestine, get mature into adult (AD) and produce new born larva (NBL). NBL migrate to striated muscle by circulatory system and become infective ML. Excretory and secretory products (ESPs) may play an important role in the developmental and invasion process of infective larva [7,8]. Immunologically, ESPs provide better opportunities to build long lasting communication between host and parasite [9].

Antigen processing and presenting through major histocompatibility complex MHC (class I & II) molecules play vital role in immune responses [10]. Basic role of gamma-interferon-inducible lysosome thiol reductase (GILT) is to help the restriction process of MHC II which catalyzes the disulfide bond reduction and to facilitate the cleavage through cellular proteases [11–13]. GILT is also named as IP30 which is expressed in many antigen presenting cells such as monocytes, B cells, macrophages and dendritic cells [11,14,15]. Researches of GILT on human, poultry, fish and other animals have been frequently reported [16]. Moreover, GILT expression may be induced by interferon- γ (IFN-γ) by activating Jak-STAT signaling pathway in endothelial cells, tumor cells and fibroblasts [17–19]. GILT is inactivated when produced, that is attached to mannose-6-phosphate receptor (M6PR) and transferred to the endosomal-lysosomal system where activated GILT is released after breakdown of endosomal pro-peptides. Mature GILT remains in lysosomes and late endosomes and shows optimum reductase activity at acidic pH in these parts [8,11,18]. GILT protein has a signature sequence (CQHGX2ECX2NX4C), an activity sequence motif (CXXC), potential glycosylation site and multiple conservative cysteine residues [20]. Previously, GILT has been reported in T cell-activated negative regulation [19], extracellular antigen neutralization and cleaning of cell debris [17].

In this study, GILT from *T*. *spiralis* (Tsp-GILT) was cloned, analyzed the sequence signature of Tsp-GILT to predict 3D structure and expressed in pET-32a. Furthermore, a soaking method was used to knockdown expression of the Tsp-GILT gene using three siRNA targeting different regions of the gene and infectivity of effective siRNA was evaluated on different developmental stages of *T*. *spiralis*.

## 2. Materials and methods

### 2.1 Ethical statement

All experimental protocols were approved by the Science and Technology Agency of Jiangsu Province (Approval ID: SYXK (SU) 2010–0005).

### 2.2 Collection of parasites

The isolate of *T*. *spiralis* (ISS534, from pig in Nanyang city, Henan Province, China) used in this study was maintained by serial passage in ICR mice every 6–8 months. MLs were recovered from *T*. *spiralis* infected mice by euthanizing at 35 post infection day, the carcasses was digested artificially using digestion solution (0.33% pepsin 1% HCl) at 43°C [5,21]. MLs were collected after 2 hours and cultured in RPMI-1640 supplemented with 20% heat-inactivated fetal bovine serum (FBS), penicillin (500 units/mL) and streptomycin (500 mg/mL) at 37°C with 5% CO_2_. To collect the adult worms, mice were infected with MLs by gavage. At 5 days post infection mice were euthanized to collect intestines. Longitudinally cut intestines were tiled on three layers of gauze in normal saline beaker, covered with aluminum file and incubated at 37°C. After 4 hours of incubation, adult worms were shed at the bottom of beaker. To collect the NBL, adult worms were cultured at 37°C with 5% CO_2_ in RPMI-1640 containing FBS (20%), penicillin (500 units/mL) and streptomycin (500 mg/mL). NBL were reproduced and separated from adults by 300-mesh steel sieve [22].

### 2.3 Molecular cloning and sequence analysis of Tsp-GILT

To clone the Tsp-GILT, total RNA was extracted from ML using TRIzol reagent (Invitrogen, Shanghai, China) [23]. The cDNA was transcribed as per manufacturer’s instructions of cDNA Kit (Takara Biotechnology, Dalian, China) and stored at -20°C till use. The ORF (open reading frame) of Tsp-GILT was amplified by RT-PCR (reverse transcription-polymerase chain reaction) using specific primers (S1 Text: P1F and P1R) based on the CDS (conserved domain sequences) of *T*. *spiralis* (Gene bank: XM_003381871.1). Gel Extraction Kit (Omega Bio-tech, Norcross, GA, USA) was used according to instructions of manufacturer to purify the PCR products and followed by ligation into cloning vector, pMD19-T (Takara, Dalian, China). Transformation of recombinant plasmid (pMD19-T/GILT) into *E*. *coli* DH5α strain (Invitrogen Biotechnology, Shanghai, China) was performed and cultured in ampicillin containing Luria Bertini (LB) medium. The recombinant plasmid, pMD19-T/GILT, was identified by restriction enzyme (*Bam*H I & *Hind* III) digestion. The positive clone was confirmed by sequencing (Invitrogen Biotechnology).

The signal peptide was predicted by signal P4.1. The multiple sequence alignment of GILTs amino acid was created with Clustal X program. The phylogenetic tree was constructed with MEGA (ML algorithm, bootstrap = 100 verifying the reliability). A homologous modeling of *Homo sapiens*, *Sus scrofa* and *T*. *spiralis* was carried out using Chimera Graphics visualized 3D model, Phyre2 web service and crystal structure of oxidoreductase disulfide bond (C3C7MB) as template. Additionally, GILT proteins of different species (n = 13) were used to construct a phylogenetic tree to determine the position of Tsp-GILT in evolution (S2 Text).

### 2.4 Expression and purification of recombinant Tsp-GILT

Another PCR was performed to get the mature peptide coding sequence with another pair of primer (S1 Text: P2F and P2R). The PCR product was purified, digested by *Bam*H I and *Hind* III, and sub-cloned into pET32a (+). Recombinant plasmid (pET-32a (+)/Tsp-GILT) was transformed into *E*. *coli* BL21 and cultured in ampicillin (100 μg/mL) containing LB at 37°C. Protein was expressed by adding 1 mM isopropyl-β-d-thiogalactopyranoside (IPTG; Sigma-Aldrich, Shanghai, China) as described previously [24]. The recombinant Tsp-GILT protein was purified according to the manufacturer’s instructions using Ni^2+^-nitrilotriacetic acid (Ni-NTA) column and refolded by renaturation buffer (20 mmol/l Tris-Cl, 500 mmol/l NaCl, 1 mmol/l GSH, 0.1 mmol/l GSSG, pH 8.0) containing different concentrations of urea (8, 6, 4, 2, 0 M) [25]. The rTsp-GILT protein was detoxified using Endotoxin Removal Kit (GeneScript, Piscataway, USA) as described previously [26].

### 2.5 Generation of polyclonal antibodies

Six female Sprague Dawley (SD) rats (body weight 150–160g) were purchased from the Experimental Animal Center of Jiangsu, P. R. China (Qualified Certificate: SCXK 2008–0004) and kept under controlled conditions at Animal house of Nanjing Agricultural University to generate polyclonal antibodies as described previously [27]. Firstly, SD rats were divided randomly into two groups, experimental group (n = 3) and control group (n = 3) to generate polyclonal antibodies against rTsp-GILT. Equally mixed rTsp-GILT protein (0.3mg) with Freund’s complete adjuvant was subcutaneously injected to experimental group. Three more doses of protein mixed equally with Freund’s incomplete adjuvant were injected with 2 weeks interval. Sera samples from control group and experimental group were collected after 10 days of last immunization and stored at -80°C till further use.

### 2.6 Thiol reductase activity of recombinant Tsp-GILT

In this study, dithiothreitol (DTT) was used as GILT requires the addition of a reducing agent for activity [28]. Thiol reductase activity of the recombinant Tsp-GILT (rTsp-GILT) was analyzed according to a previous method [29]. Briefly, rTsp-GILT was diluted in acidic buffer (0.1% TritonX-100 in 100 mM NaCl, 50 mM acetate, pH 4.5) and pre-activated by adding DTT to 10 mM final concentration at 37°C for 10 min. Rabbit IgG (Diluted in acidic buffer) was added to pre-activated rTsp-GILT and incubated at 37°C for 15 min, then the reaction was stopped by adding 5 × non-reducing SDS loading buffer. Finally, IgG reduction into heavy and light chains was analyzed by non-reducing SDS-PAGE.

### 2.7 Localization of Tsp-GILT

*T*. *spiralis* MLs were suspended in optimal cutting temperature compound (SAKURA, Torrance, CA, USA) after fixation on glass slides (poly-l-lysine hydrobromide) with 4% formaldehyde-0.2% glutaraldehyde in PBS for 45 min and blast freeze in liquid nitrogen. parasites were cut into pieces (10-μm thickness) through cryotome (CM1950, Wetzlar, Germany) and washed with PBS. The slides were treated with 5% BSA to block non-specific bindings followed by incubation with 1:300 dilutions of rat-anti-rTsp-GILT antiserum (experimental group) and normal rat serum (control group) as first antibody at 37°C. After 2 h incubation, slides of both groups were incubated with 1:3,000 dilutions of Cy3-labeled Goat Anti-Rat as second antibody (Beyotime, Shanghai, China) at 37°C for 1h. DAPI (diamidino-2-phenylindole) was used to stain the nucleus of worm cells and anti-Fade Fluoromount Medium (Beyotime, Shanghai, China) was used before observing under confocal laser scanning microscope.

### 2.8 Transcription level of Tsp-GILT mRNA

Quantitative reverse transcriptase PCR (QRT-PCR) was performed to evaluate gene transcription level of Tsp-GILT using SYBR-Green I qPCR Master Mix kit and ABI 7500 to evaluate target gene expression in 2^−△△Ct^ method [30]. Initially, total RNA was extracted using TRIzol method (Invitrogen, Shanghai, China) [23] from various stages of *T*. *spiralis* (NBL, ML and AD). Moreover, cDNA was synthesized from RNA using Thermo-Script RT and Oligo (dT) (Invitrogen, Foster, CA, USA) according to the manufacturer’s instructions. Different primers of Tsp-GILT and Tubulin (housekeeping gene of *T*. *spiralis*) were designed using IDT web (http://sg.idtdna.com/primerquest/Home/Index) (S1 Text: P3Q-F, P3Q-R, Tubulin-F and Tubulin-R).

### 2.9 Effects of mouse IFN-γ on parasite Tsp-GILT mRNA transcription

QRT-PCR was performed to evaluate the stimulatory effects of different concentration of mouse IFN-γ (Genscript, China) on transcription level of Tsp-GILT *in vitro*. Firstly, MLs were cultured in RPMI-1640 (supplemented with 20% FBS, penicillin and streptomycin) with PBS (control) and different concentrations of mouse IFN-γ (250, 500, 750, 1000, 1250, 1500 ng/mL) at 37°C with 5% CO_2_. After 24 h incubation, PBS and IFN-γ treated MLs were collected and cDNA was synthesized after extraction of total RNA as described above.

### 2.10 siRNAs preparation

Three specific siRNA were designed based on Tsp-GILT gene sequence. Each siRNA targeted different part of Tsp-GILT coding sequence (S3 Text). siRNA1, 2 & 3 and control siRNA were chemically synthesized by Ribobio, Guangzhou, China. Immunofluorescence assay was performed to confirm the transformation of siRNA into *T*. *spiralis* ML and NBL as described previously [27]. Initially, MLs and NBLs were cultured in RPMI-1640 (supplemented with penicillin 500 units/mL and streptomycin 500 mg/mL) with Cy3 labeled control siRNA (Beyotime, Jiangsu, China) in 12-wells culture plates (500 MLs/500 ul) at 37°C with 5% CO_2_ for 36 h. Followed by adding 20% FBS and incubating for another 24 h. Then MLs and NBLs were fixed on glass slides (on poly-L-lysine coated) with 4% phosphate-buffered paraformaldehyde at room temperature for 30 min, the fluorescence was visualized by confocal microscope.

### 2.11 Knockdown of Tsp-GILT gene expression in ML and NBL

To knockdown Tsp-GILT gene expression, soaking method was used [4]. MLs were cultured in RPMI-1640 (supplemented with penicillin 500 units/mL and streptomycin 500 mg/mL) with siRNA1, siRNA2 and siRNA3 in 12-wells culture plates (500 MLs/500 ul) at 37°C with 5% CO_2_. After 36 h incubation, 20% FBS was added and further incubated for 24 h. PBS and control siRNA were used as controls. TRIzol Reagent (Sango Biotech, China) was used to extract total RNA from siRNA-treated parasites according to the manufacturer’s instructions. Subsequently, cDNA was synthesized as per discussed above and QRT-PCR was performed to evaluate Tsp-GILT gene transcription using primers listed in S1 Text (P3Q-F, P3Q-R, Tubulin-F and Tubulin-R). The most effective QRT-PCR-identified siRNA was selected for knockdown of Tsp -GILT in ML and NBL.

To estimate the survival rate of ML and NBL *in vitro* after suppression of Tsp-GILT expression, soaked siRNA2, control siRNA and PBS treated MLs and NBLs were cultured in RPMI-1640 containing 20% FBS, 500 units/mL penicillin and 500 mg/mL streptomycin, at 37°C with 5% CO_2_. After 24 h incubation, MLs and NBLs contained culture solution was well blended, absorbed and dripped on slides and viability of larvae was observed under inverted microscope. The larvae which was active in wriggling motion was considered as live, while the larva which was not active or not having straight “C” shape was considered as non-viable and larva was considered as dead if larva still remain non active for 6h at 37°C [31].

### 2.12 Evaluation the infectivity of effective siRNA treated ML

Thirty mice were purchased from the Experimental Animal Center of Jiangsu, P. R. China (Qualified Certificate: SCXK 2008–0004) and kept under controlled conditions at Animal house of Nanjing Agricultural University. To evaluate the infectivity of effective siRNA-treated *T*. *spiralis*, mice were divided randomly into three groups (n = 10 for each group), experimental group, control group and PBS group were infected orally with 500 MLs treated with siRNA2, control siRNA or PBS, respectively. Five days post infection, five mice from each group were euthanized by head dislocation, Adult worms were collected from mice intestines by the method discussed above. Subsequently, adult worms were dripped in 12-well culture plate and counted one by one. Moreover, to estimate and collect the number of NBL, 100 adult worms from each group were cultured separately in RPMI-1640 containing FBS (20%), penicillin (500 units/mL) and streptomycin (500 mg/mL) at 37°C with 5% CO_2_ for 24 h. Consequently, NBLs were separated from adult worms by 300-mesh steel sieve from each group and the number of NBLs were counted. Remaining five mice from each group were euthanized at 35 DPI, and total number of next generation MLs were collected from each group by digestion method.

### 2.13 Evaluation the infectivity of effective siRNA treated NBL

Fifteen mice were divided into 3 groups (n = 5 for each group) and infected through tail vein injection with 20000 NBLs treated by siRNA2, control siRNA and PBS group, respectively. At 35 DPI, mice were euthanized by head dislocation and MLs were collected from mice of each group by artificial digestion as described above. ML reduction was calculated from the group treated with siRNA2 and compared with control siRNA treated.

### 2.14 Statistical analysis

Statistical analyses were performed using GraphPad Prism 7.0 (GraphPad Prism, USA). All data obtained from the above experiments were displayed as mean ± SEM. One way ANOVA followed by Tukey’s *post-hoc* test was employed to compare the variances between groups and considered statistically significant at *p < 0.05, **p <0.01, ***p <0.001.

## 3. Result

### 3.1 Identification and analysis of Tsp-GILT

**Fig 1:**
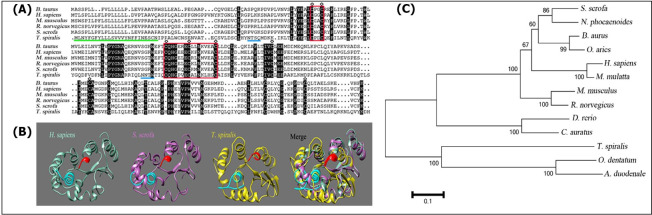
**A**: Analysis and multi-alignment of rTsp-GILT sequence with GILT of other known species. Identical amino acids among all sequences are indicated by black background. The active-site motif of CXXC and GILT signature sequences are highlighted in red boxes. The predicted signal peptide is marked with green underlining. The glycosylation sites are indicated by blue underlining. The conserved cysteines sites were marked by black ◇. **B**: Alignment of the predicted 3D structure of *T*. *spiralis* with *H*. *sapiens* and *S*. *scrofa*. **C**: The phylogenetic tree of 13 GILT proteins.

The cDNA of Tsp-GILT was successfully amplified by RT-PCR. Its ORF spans 801 base pairs, encodes a protein of 266 amino acids, with a calculated molecular weight about 29 kDa. Primary structure analysis of Tsp-GILT confirmed the presence of main characteristics of GILT protein including active site CXXC motif, signature sequence (CQHGX2ECX2NX4C), and 9 conserved cysteines sites (Fig 1A).

The 3D structures of GILTs (*Homo sapiens*, *Sus scrofa* and *T*. *spiralis*) were predicted by the comparative modeling using crystal structure of oxidoreductase disulfide bond (C3C7MB) as template. The tertiary structures of *Homo sapiens* GILT, *Sus scrofa* GILT and *T*. *spiralis* GILT were moderately similar in spatial position and shape (Fig 1B).

Phylogenetic analysis among GILT of different species revealed that all sequences were grouped into two well-defined clusters of vertebrates and invertebrates (Fig 1C).

### 3.2 Expression and thiol reductase activity of recombinant Tsp-GILT

**Fig 2:**
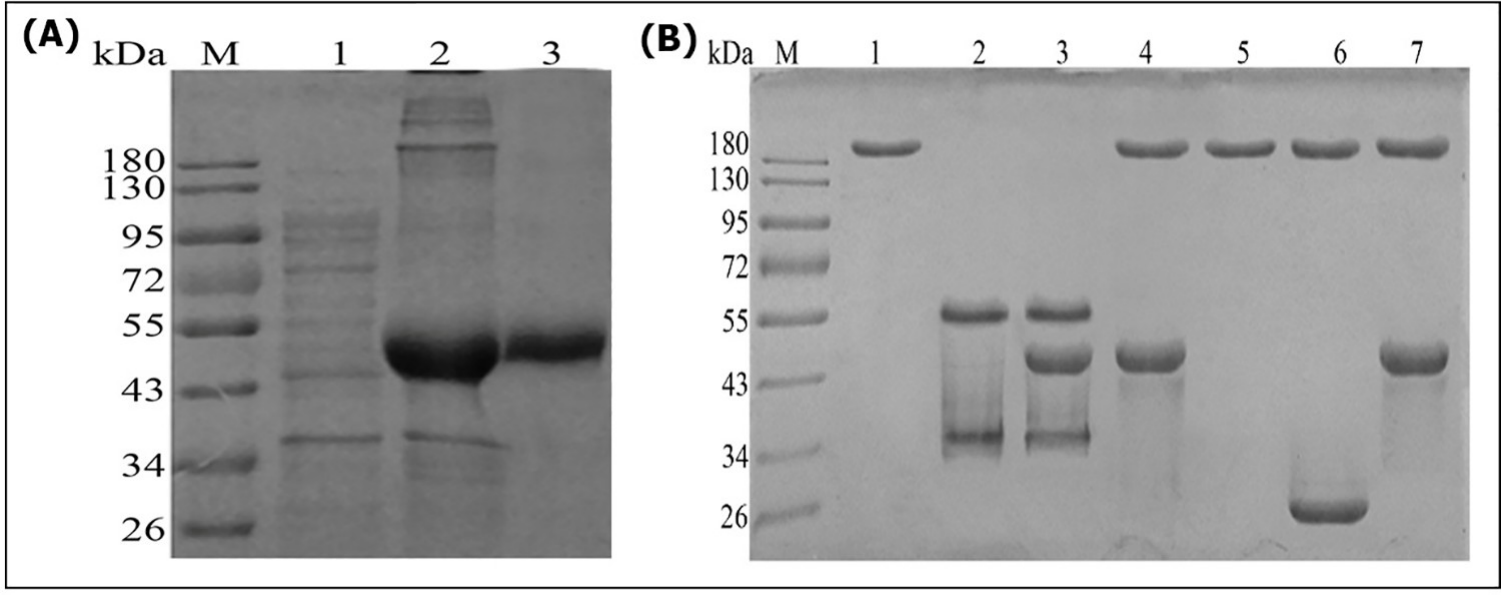
**A**: Purification of Recombinant protein. M: Protein molecular weight Marker; Lane 1: Supernatant of recombinant *E*. *coli* lysis; Lane 2: Inclusion body from recombinant *E*. *coli*; Lane 3: Purified recombinant protein. **B**: Tsp-GILT exhibits thiol reductase activity in vitro. M, protein molecular weight marker; Lane 1: Rabbit IgG treated without DTT at pH 7.0 as negative control; Lane 2: Rabbit IgG treated with 50 mM DTT at pH 7.0 as positive control; lane 3:Rabbit IgG incubated with Tsp-GILT (activated by 10 mM DTT at pH 4.5); Lane 4:Rabbit IgG incubated with Tsp-GILT (without DTT at pH 4.5); Lane 5: Rabbit IgG incubated without Tsp-GILT, with 10 mM DTT at pH 4.5; Lane 6: Rabbit IgG incubated with pET32a tag protein (treated with 10 mM DTT at pH 4.5); Lane 7: Rabbit IgG incubated with Tsp-GILT (treated with 10 mM DTT at pH 7.0).

The recombinant Tsp-GILT was expressed in *Escherichia coli* BL21 (DE3). The His6-tagged Tsp-GILT (46 kDa) was efficiently expressed and purified with denaturation-renaturation procedure followed by Ni^2+^ affinity chromatography (Fig 2A).

As a positive control, the denatured IgG (about 180 kDa) was reduced into H chain (55 kDa) and L chain (34 kDa) with 50 mM DTT after an hour incubation at 37°C, pH 7.0 (Fig 2B, lane 2). While, at pH 4.5, denatured IgG was reduced into H chain and L chain by activated Tsp-GILT with 10 mM DTT (Fig 2B, lane 3). In contrast, un-activated Tsp-GILT (without incubation with 10 mM DTT) could not reduce the denatured IgG into H chain and L chain at both pH 4.5 and pH 7.0 (Fig 2B, lane 4 and 7). The result indicates that Tsp-GILT exhibits thiol reductase activity in acidic environment and requires the addition of a reducing agent (low concentration of DTT) for activity *in vitro*.

### 3.3 Immunohistochemical study of Tsp-GILT in ML

**Fig 3:**
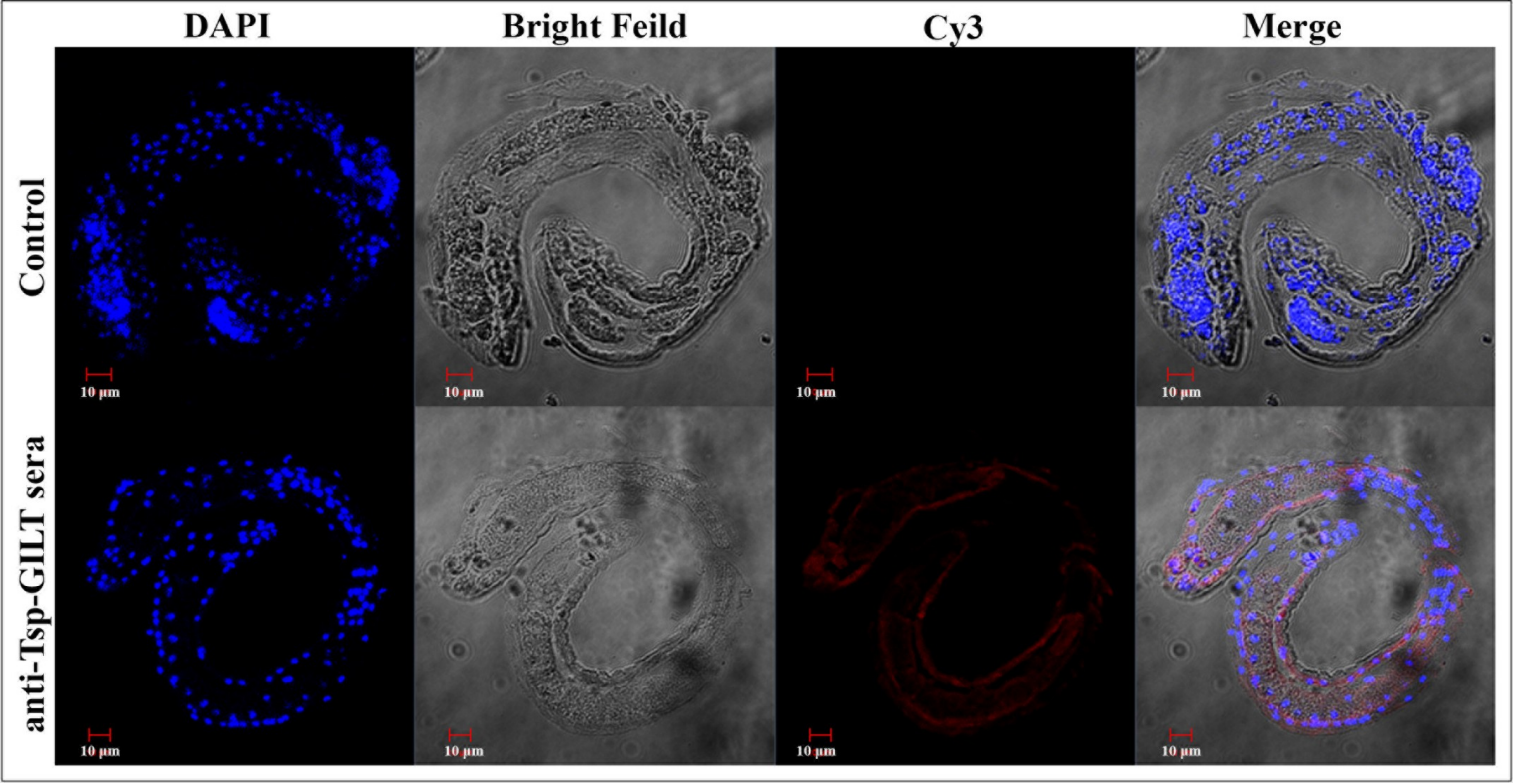
Localization of Tsp-GILT in *T*. *spiralis* ML by immunofluorescence assay. First line is negative control group, second line is experience group. Nuclei were stained with DAPI (blue) and target protein with Cy3 (red). No red fluorescence was observed in control.

A straight “C” shape body of *T*. *spiralis* ML was shown in Fig 3. Blue spots Clusters inside the body of ML indicated nuclei along the gut structure. The IFA results revealed that Tsp-GILT might be localized outer and inner surface of the membrane as well as in gut section when incubating with anti-Tps-GILT rat sera. While no protein labeling was seen in control section.

### 3.4 Tsp-GILT transcription

**Fig 4:**
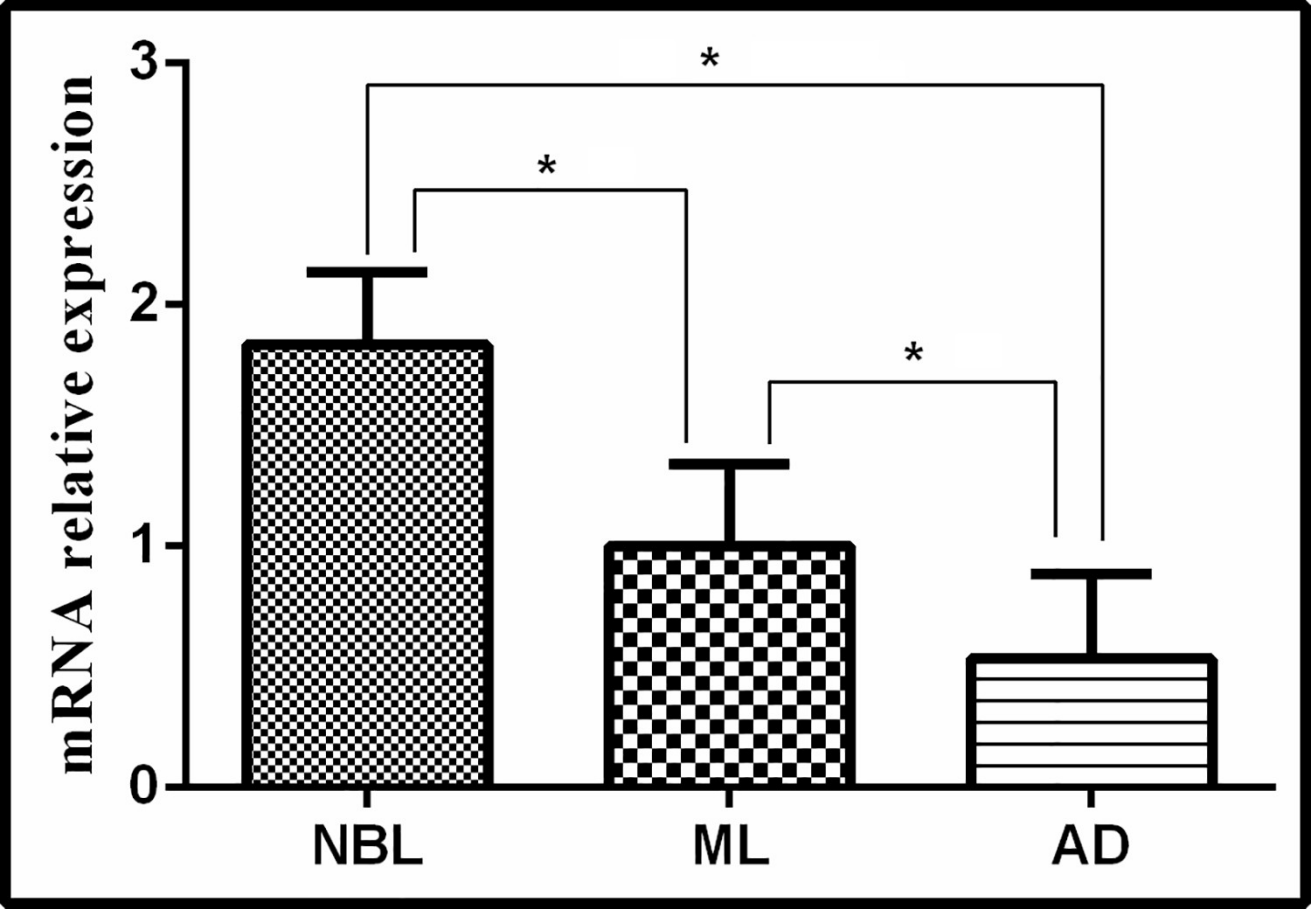
Transcription level of Tsp-GILT by QRT-PCR during various development stages of *T*. *spiralis*. NBL: Newborn Larva (relative fold = 1.80); ML: Muscle Larva(relative fold = 1); AD; Adult worms(relative fold = 0.58). Significant differences were observed as *P < 0.05.

QRT-PCR was performed to analyze the transcription level of Tsp-GILT in various development stages of *T*. *spiralis*. Statistical analysis revealed that Tsp-GILT showed significantly higher (P<0.05) transcription level in NBL (Relative fold = 1.80) as compared to AD (Relative fold = 0.58) and ML (Relative fold = 1). The relative expression in AD was significant (P<0.05) lower than that in ML (Fig 4).

### 3.5 Effects of mouse IFN-γ on Tsp-GILT expression

**Fig 5:**
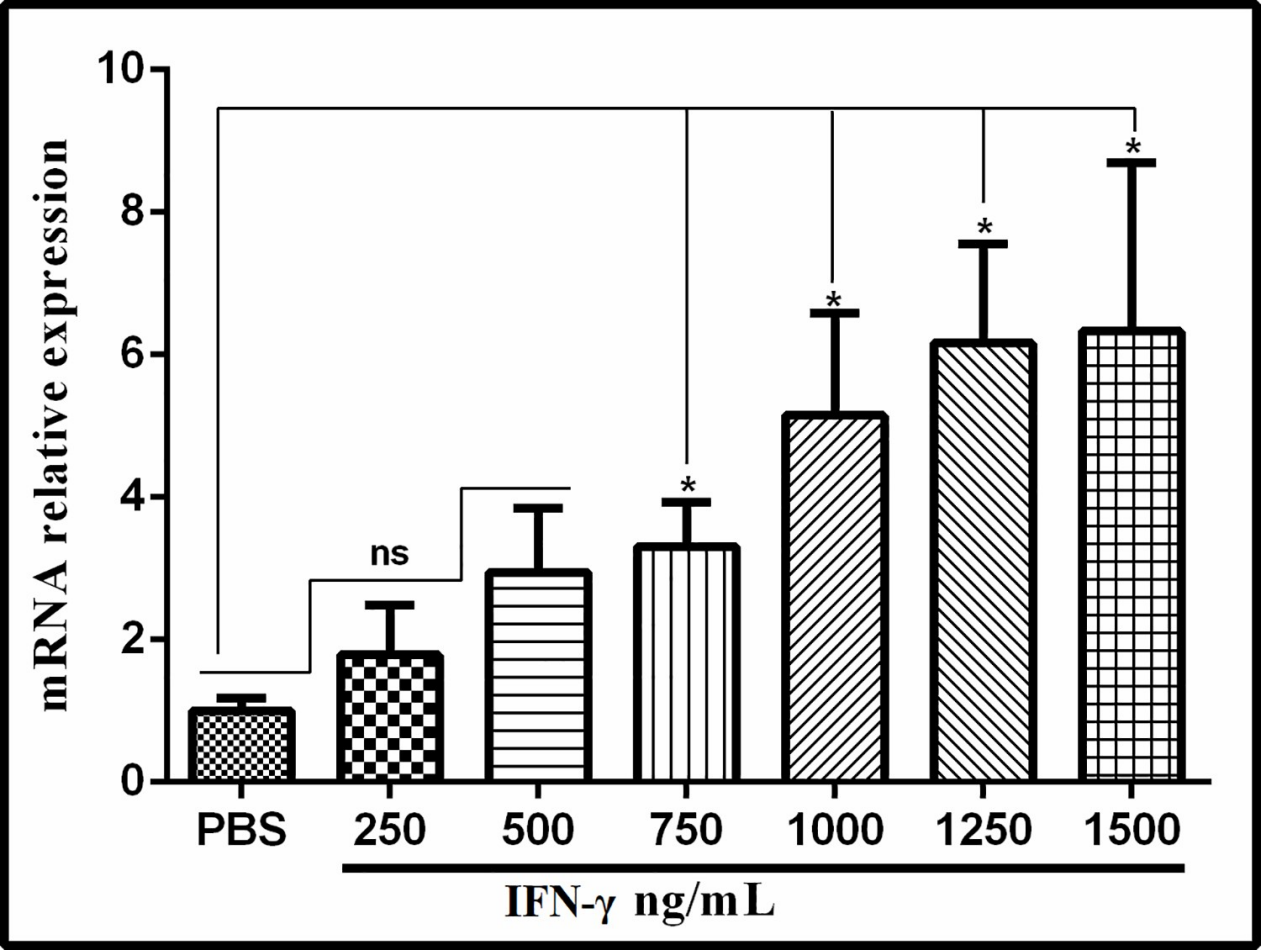
QRT-PCR analysis of Tsp-GILT expression during muscle larval stage treated with different concentration of IFN-γ and PBS (control). The significant level was set at *P < 0.05, and ns non-significant compared with control group.

QRT-PCR was performed to analyze the Tsp-GILT expression during muscle larval stage treated with different concentration of mouse IFN-γ. It was noted that expression of Tsp-GILT was significantly increased as compared to PBS group when MLs were incubated with different concentrations of mouse IFN-γ (Fig 5). Significant difference was observed between PBS and 750ng/mL to 1500ng/mL (P<0.05). While no significant difference was observed between 250ng/mL and 500ng/mL (P>0.05), indicating that the GILT expression level was saturated under 1000-1250ng/mL. These findings revealed that Tsp-GILT expression can be up-regulated by stimulation of mouse IFN-γ.

### 3.6 Knockdown of GILT expression in ML and NBL

**Fig 6:**
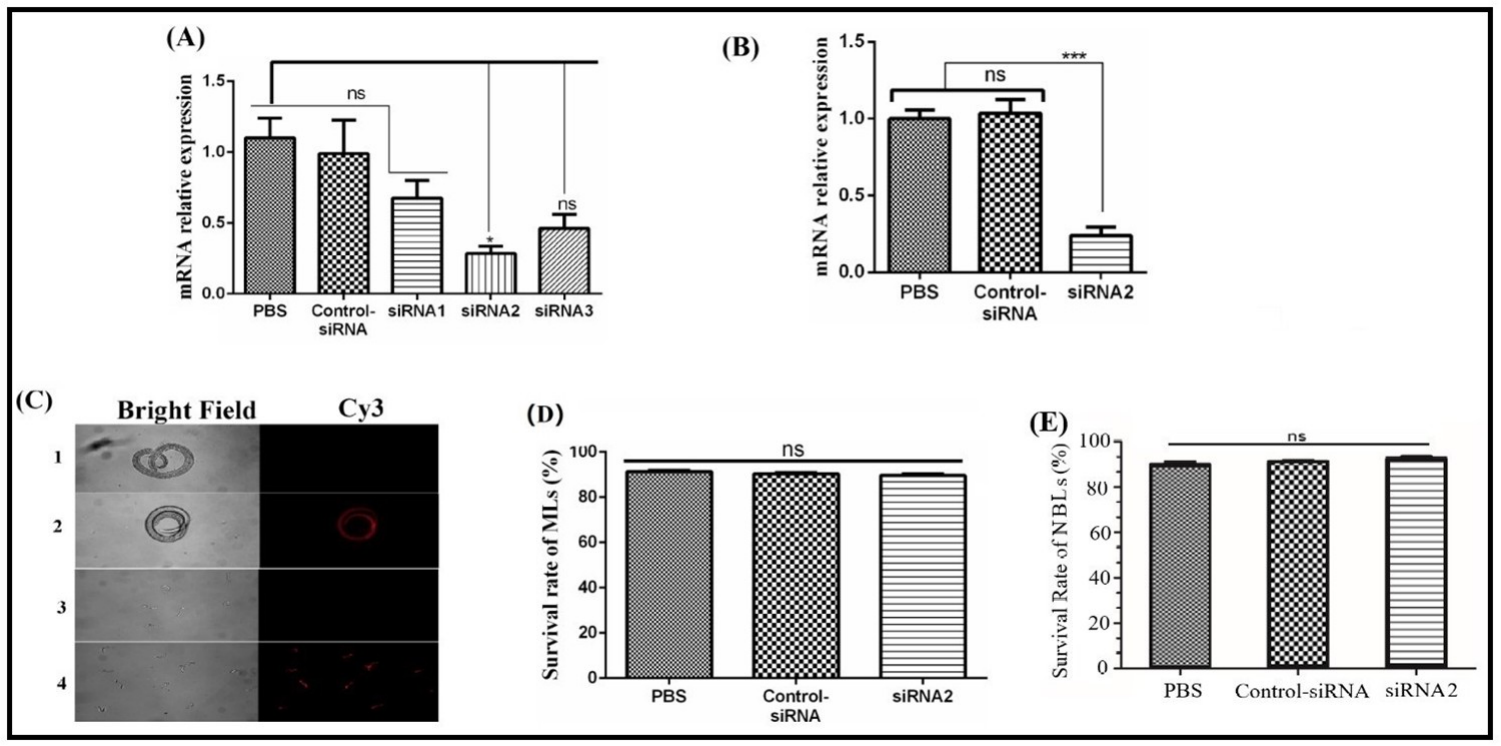
Knockdown of Tsp-GILT gene in ML and NBL. **A:** Tsp-GILT mRNA transcription level of MLs treated with siRNA1, siRNA2, siRNA3, control siRNA and PBS. **B:** Tsp-GILT mRNA transcription level of NBLs treated with siRNA2, control siRNA and PBS. **C:** Immunofluorescence assay of siRNA treated ML and NBL. Row 1: PBS treated ML; Row 2; Cy3 labeled control siRNA treated ML. Row 3: PBS treated NBL; Row 4; Cy3 labeled control siRNA treated NBL. Control siRNA treated ML and NBL showed transformation indicating red color. While, no color was observed in both control groups. **D:** Survival rate (%) of ML and NBL **(E)** *in vitro* after suppression of Tsp-GILT expression by siRNA2. The significant level was set at *P < 0.05, and ns non-significant compared with control group.

MLs were treated with siRNA1, siRNA2, siRNA3, control siRNA and PBS for 60 h. QRT-PCR analysis showed significant reducing level (P < 0.05) of Tsp-GILT mRNA in siRNA2-treated MLs. While, no significant difference (P > 0.05) was observed between siRNA1, siRNA3-treated MLs, control siRNA and PBS-treated MLs groups (Fig 6A). The most efficient QRT-PCR-identified siRNA (siRNA2) was selected to treat NBL. It was found that Tsp-GILT mRNA in siRNA2 treated NBLs was down-regulated by 76.75% when compared with control siRNA (Fig 6B). Moreover, immunofluorescence assay was performed to confirm the transformation of siRNA into *T*. *spiralis* ML and NBL. Both siRNA-treated ML and NBL showed successful transformation indicating red color. While no color indication was observed in control groups (Fig 6C). Furthermore, survival rate (%) of siRNA2, control siRNA and PBS treated MLs were observed as 92.8 ± 1.84, 93.4 ± 0.55 and 94.4 ± 1.14, respectively. No significant difference (P > 0.05) was observed between all groups (Fig 6D). No significant difference was found in the survival rate of siRNA2 treated NLs when compared with control siRNA or PBS (Fig 6E).

### 3.7 Infectivity of siRNA2 treated ML

**Fig 7:**
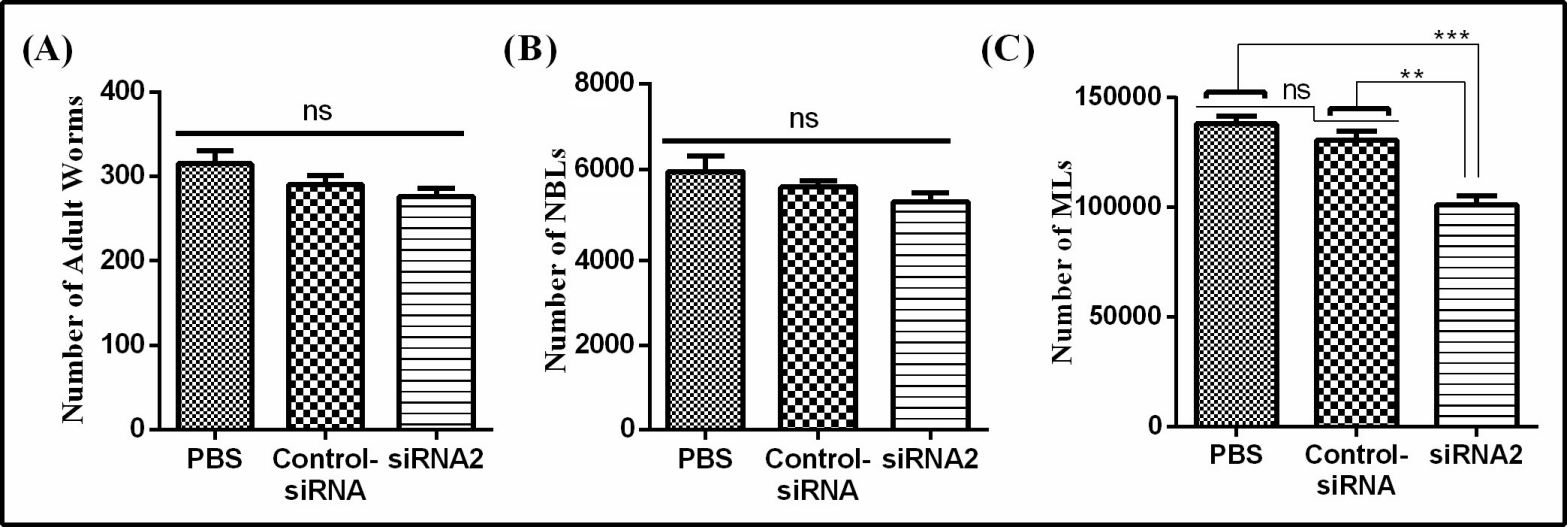
Infectivity of siRNA2 treated ML. A: Number of adult worms. B: Number of NBLs. C: Number of next generation MLs. The significant level was set at **P < 0.01, ***P < 0.001, and ns non-significant compared with control group.

No statically significant difference in the total number adult worms was observed (Fig 7A) between 276.4 ± 22.14 (siRNA2), 290.4 ± 24.40 (control siRNA) and 315 ± 35.13 (PBS). The number of NBL produced by adults in siRNA2, control siRNA and PBS treated groups were 5485 ± 515.08, 5652 ± 333.40 and 5887 ± 696.63 respectively (Fig 7B), no statically significant difference was observed among all groups (P>0.05).

Counting of next generation of MLs (ngMLs) from mice infected by siRNA2, control siRNA and PBS treated MLs was performed. Significant difference in number (22.6% reduction, P < 0.05) of ngMLs was observed between siRNA2 group (101280 ± 8036.01) and control siRNA group (130880 ± 9005.11) (Fig 7C). While, numbers of ML counted from control siRNA group and PBS group (138040 ± 8241.84) were non-significant to each other. The result indicated that siRNA2 reduced the production of MLs in new generation by specific suppression of Tsp-GILT mRNA expression.

### 3.8 Infectivity of siRNA2 treated NBL

**Fig 8:**
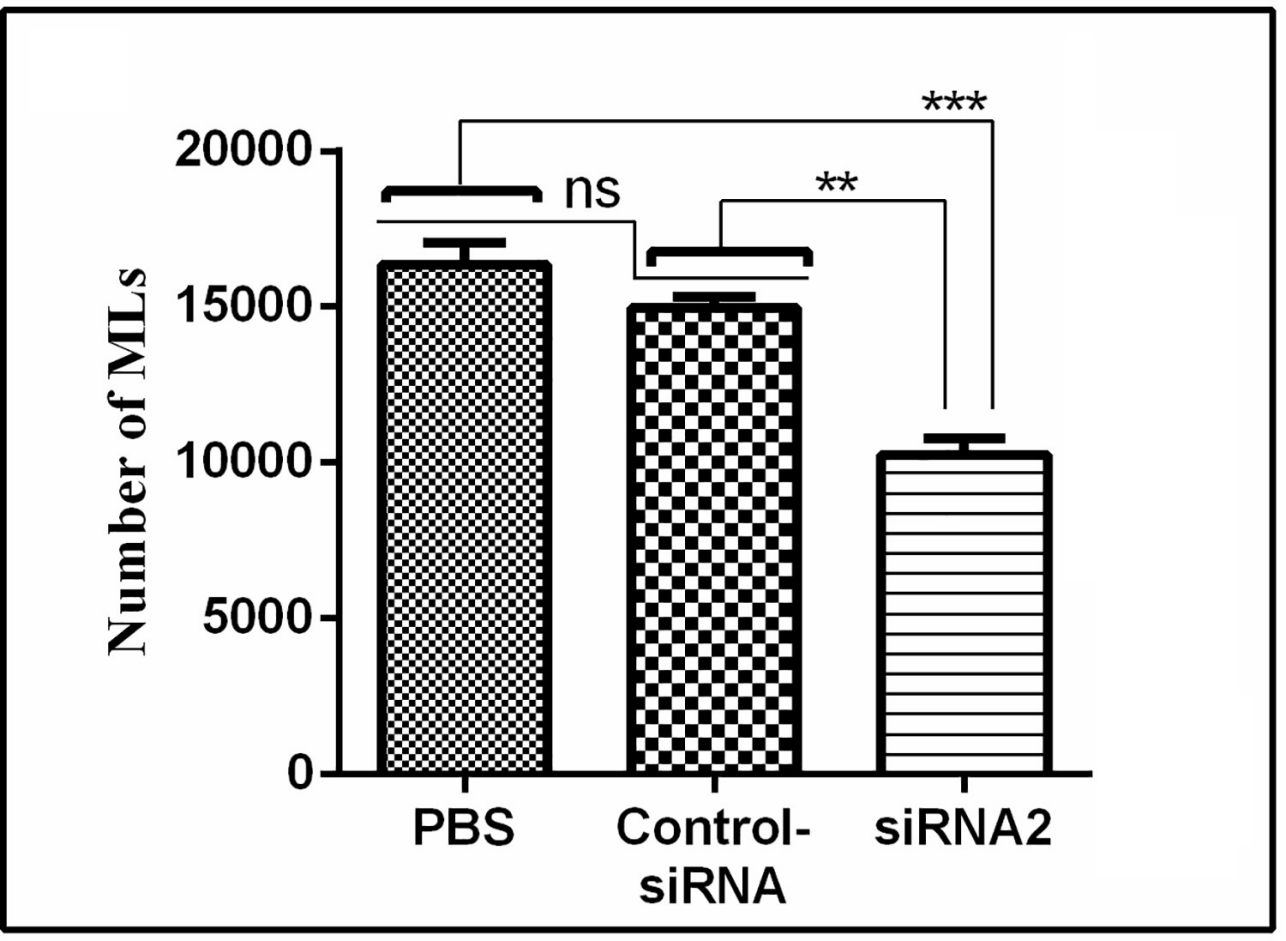
Infectivity of siRNA2 treated NBL. Number of ML population developed from siRNA2 treated NBLs. The significant level was set at **P < 0.01, ***P < 0.001, and ns non-significant compared with control group.

NBLs treated with siRNA2, control siRNA and PBS were injected in mice through tail vein and MLs were collected at 35 DPI. The number of MLs collected from siRNA2, control siRNA and PBS treated groups were 10264 ± 1201.87, 15160 ± 1089.54 and 16360 ± 1564.61, respectively. Significant difference in number of MLs between siRNA2 treated group and control siRNA or PBS treated group (P < 0.05) was found (Fig 8). These results indicated that suppression of Tsp-GILT mRNA in NBL causes 32.3% reduction of ML.

## Discussion

Specific RNAi gene knockdown was first and developed in *C*. *elegans* for analysis of functional genomics [1]. RNAi is an important method of gene function study in molecular biology research. It has been widely used in gene function and identification of parasite including single celled protozoon and nematode [32]. RNAi was successfully performed in parasitic nematode *Nippostrongylus brasiliensis* [33]. In this study, the cDNA sequence of GILT from *Trichinella spiralis* was cloned to analyze its molecular characterization, expression profile, localization, and the thiol reductase activity of recombinant TSP-GILT protein. Molecular characterization of GILT has been reported in different organisms such as soft-shelled turtle [34], silver carp [35], guinea pig [36] and cuttlefish *Sepiella japonica* [37]. As described in this study, Tsp-GILT protein possesses all the main typical features of known GILT proteins. The sequence alignment indicated that Tsp-GILT showed a high homology with other know GILTs on functional domain sequence suggests that they may have a similar function in immunology. From the phylogenetic tree, it is seen that these represent the relationship of GILTs among 13 species and suggest them of different evolution direction. Therefore, these analyses provide evidence of GILT family genes that have been originated from a common ancestral gene during evolution but still need further extending with the identification of new GILT genes. As there is no real crystal structure of GILT, in this study the 3D structures of GILTs of several species were predicted by homology modeling based on the crystal structures of disulfide oxidoreductase.

To study the activity of the Tsp-GILT protein, we expressed the ORF of Tsp-GILT in *E*. *coli* with pET32a plasmid. We got the activated soluble recombinant protein through purification and refolding. In the early studies, GILT proved to be capable of catalyzing the reduction of the inter chain disulfide bonds intact IgG. Recently, more and more studies had confirmed the thiol reductase activity of GILT in many species. Tsp-GILT could also reduce IgG into H and L chains, and the reduction occurred at pH 4.5. It is important to note that the reducing power of DTT is limited to pH values above 7, since only the thiolate form is reactive [11]. This recombinant Tsp-GILT expressed and purified from *E*. *coli* was active, suggesting that glycosylation is not essential for Tsp-GILT thiol reductase activity. However, we could not exclude the possibility that glycosylation is important for the regulation of enzyme stability or function. In contrast to thioredoxin, which is more efficient at neutral pH, GILT has maximal reductase activity at an acidic pH, consistent with its function being mediated in late endocytic compartments and lysosomes. The qRT PCR analysis revealed that Tsp-GILT was constitutively expressed in all three stages of worm, with high mRNA level observed in NBL which can contact with host immune cells and lower level in AD which live in host intestine. IFT analysis showed that Tsp-GILT was mainly expressed in those cells that are near epidermis and digestive tract of worms. Interestingly enough, those locations of worm direct were most likely to be attacked by weapons of host immune system.

The existence of IFN-γ in *T*. *spiralis* and the homology analogs were not reported in mainstream proteomic database. Therefore, we used the mouse IFN-γ to stimulate the worms *in vitro* and to observe the changes of Tsp-GILT mRNA expression. Statistical analysis suggested that after IFN-γ stimulation the expression level of the Tsp-GILT mRNA *in vitro* was significant up-regulation compared to the PBS-treated group, with the increase of concentrate of IFN-γ. The result indicted that Tsp-GILT may be involved in the worm response against host immune system.

In this study the effects of siRNA-mediated knockdown on life movement of *T*. *spiralis* were evaluated. It has been found by RNAi experiment that, the suppression of Tsp-GILT didn’t have a significant influence on the survival of ML *in vitro*, development and reproduction of adults, but the quantity of next generation of ML was reduced significantly. To confirm this phenomenon, mice were infected with NBL interfered by siRNA-2 through tail vein injection. In the case of no significance difference of life movement on parent worms but the next generation reduced. The possible explanation is that the gene silencing of parents was transported into next generation by germ cells. The result showed that, the quantity of ML was also reduced significantly. It indicated that the suppression of Tsp-GILT expression would reduce the survival of *T*. *spiralis* in the internal environment of host.

Current study revealed that Tsp-GILT, a member of the GILT family, contains the main features of know GILT in sequence, 3D structure and enzyme activity. Additionally, mRNA up-regulation of Tsp-GILT expression under the induction by mice IFN-γ suggest that Tsp-GILT is a potential function that worm against with host immune system. Moreover, the expression of Tsp-GILT in NBL is higher than other stages, and NBL is the stage in the internal environment of host. Finally, the suppression of Tsp-GILT resulted in significant decrease of the number of ML *in vivo*, but other life movement have no significant differences, indicating the Tsp-GILT possibly contribution in the viability and survival of NBL in the host. Based on the above experimental evidence, the Tsp-GILT probably plays an important role in the interaction between *T*. *spiralis* and host immune system, even in the processing of worm immune escape.

## Conclusion

Current study concluded that siRNA knockdown of gamma interferon inducible lysosomal thiol reductase from *Trichinella spiralis* significantly reduce the larval infectivity, development and survival in mice.

## Supporting information

S1 Text Primers used in this study. (DOC)

S2 Text Accession ID of all GILT protein used in this study. (DOC)

S3 Text siRNAs sequences. (DOC)
