# Supplementary material for: Trichinella spiralis: Knockdown of gamma interferon inducible lysosomal thiol reductase (GILT) results in the reduction of worm burden
Source: PLoS Negl Trop Dis. 2021 Nov 30;15(11):e0009958. doi: 10.1371/journal.pntd.0009958 (PMC8631631; doi:10.1371/journal.pntd.0009958)
Supplement: S1 Text — (DOC) [file pntd.0009958.s001.doc]

Primers used in this study

| **Name** | **Nucleotide sequence (5′-3′)** |
| --- | --- |
| P1-F | ATGCTGAATTATTTTGGCTTTTATCTGT |
| P1-R | TTATTTGTGATCTTTGTATACCTGCAAATTC |
| P2-F | CGGGATCC CCGCCAGCACTTTGG |
| P2-R | CCCAAGCTT TTATTTGTGATCTTTGTATACCTGCA |
| P3Q-F | TCCTGATGCAGCATTCTTACTT |
| P3Q-R | TCAGATCACCTTCTACGCTATTTG |
| Tubulin-F | TGCATTGGTACACTGGAGAAG |
| Tubulin-R | GCTTCCTGGTACTGCTGATATT |
