# Supplementary material for: Trichinella spiralis: Knockdown of gamma interferon inducible lysosomal thiol reductase (GILT) results in the reduction of worm burden
Source: PLoS Negl Trop Dis. 2021 Nov 30;15(11):e0009958. doi: 10.1371/journal.pntd.0009958 (PMC8631631; doi:10.1371/journal.pntd.0009958)
Supplement: S2 Text — (DOC) [file pntd.0009958.s002.doc]

**Accession ID of all GILT protein used in this study**

| **Species** | **Accession ID** |
| --- | --- |
| *Homo sapiens* | P13284 |
| *Mus musculus* | Q9ESY9 |
| *Sus scrofa* | B3SP85 |
| *Rattus norvegicus* | Q499T2 |
| *Bos taurus* | A6QPN6 |
| *Ancylostoma duodenale* | A0A0C2D594 |
| *Trichinella spiralis* | E5RZK6 |
| *Oesophagostomum dentatum* | A0A0B1T4W1 |
| *Macaca mulatta* | H9YYT0 |
| *Ovis aries* | F1ASZ5 |
| *Neophocaena phocaenoide* | X2CT61 |
| *Danio rerio* | Q5XJN2 |
| *Carassius auratus* | E7E2N8 |
