# Supplementary material for: Trichinella spiralis: Knockdown of gamma interferon inducible lysosomal thiol reductase (GILT) results in the reduction of worm burden
Source: PLoS Negl Trop Dis. 2021 Nov 30;15(11):e0009958. doi: 10.1371/journal.pntd.0009958 (PMC8631631; doi:10.1371/journal.pntd.0009958)
Supplement: S3 Text — (DOC) [file pntd.0009958.s003.doc]

siRNAs sequences

| **siRNA** | **Sense (5’-3’)** | **Antisense (5’-3’)** | **Position** |
| --- | --- | --- | --- |
| siRNA1 | GCUAGGAGAAUCCAACUUAdTdT | UAAGUUGGAUUCUCCUAGCdTdT | 304-322 |
| siRNA2 | GCAGCAUUCUUACUUUGCAdTdT | UGCAAAGUAAGAAUGCUGCdUdT | 478-496 |
| siRNA3 | GCGAAAGAAUUUGCAGGUAdTdT | UACCUGCAAAUUCUUUCGCdTdT | 765-793 |
